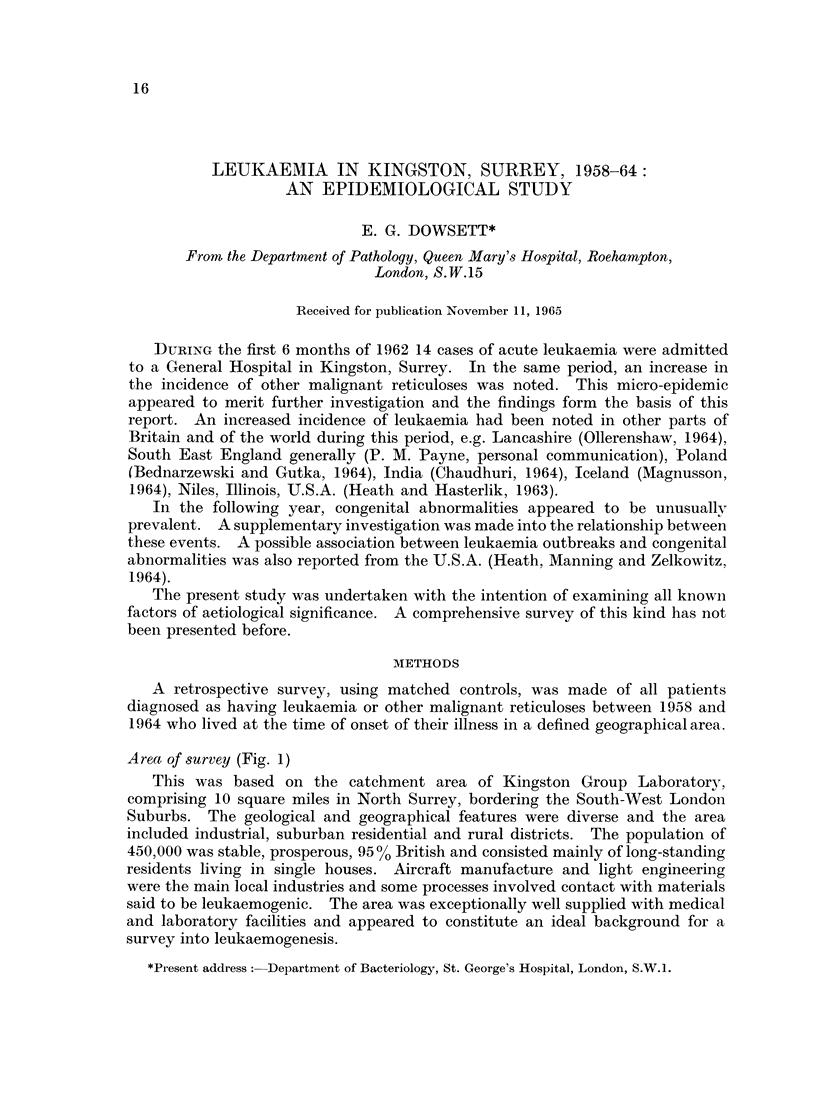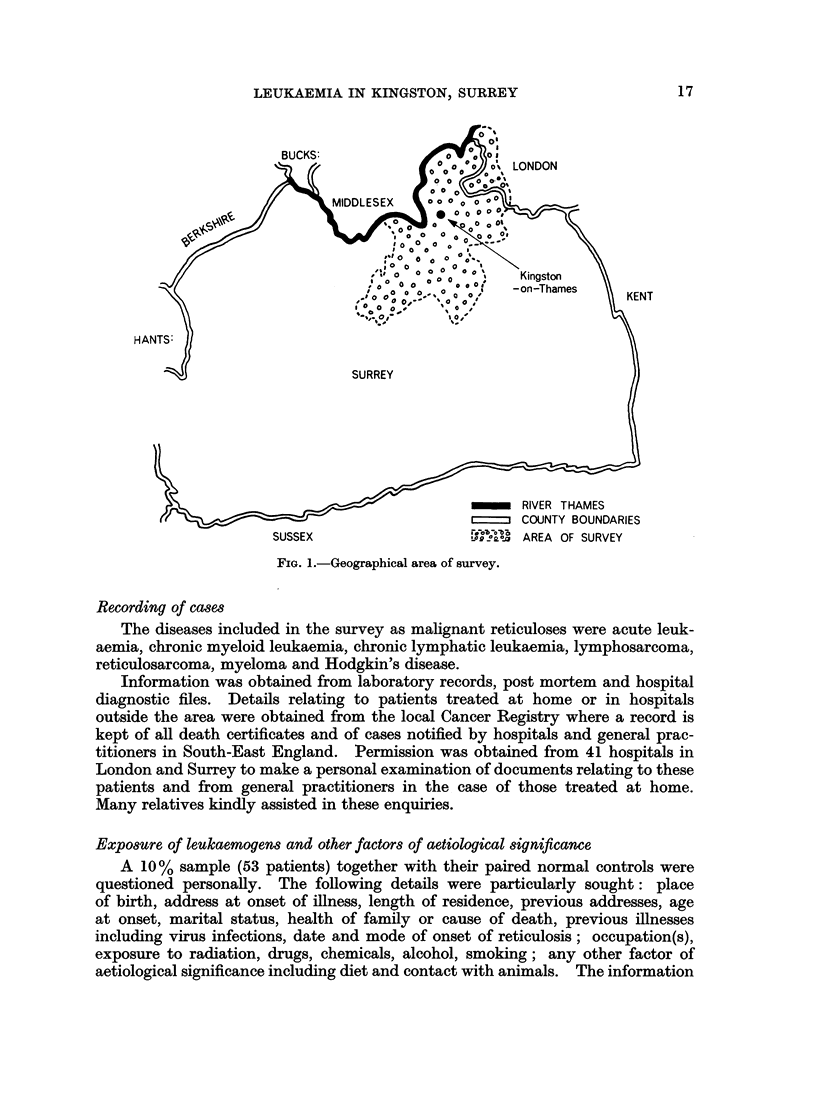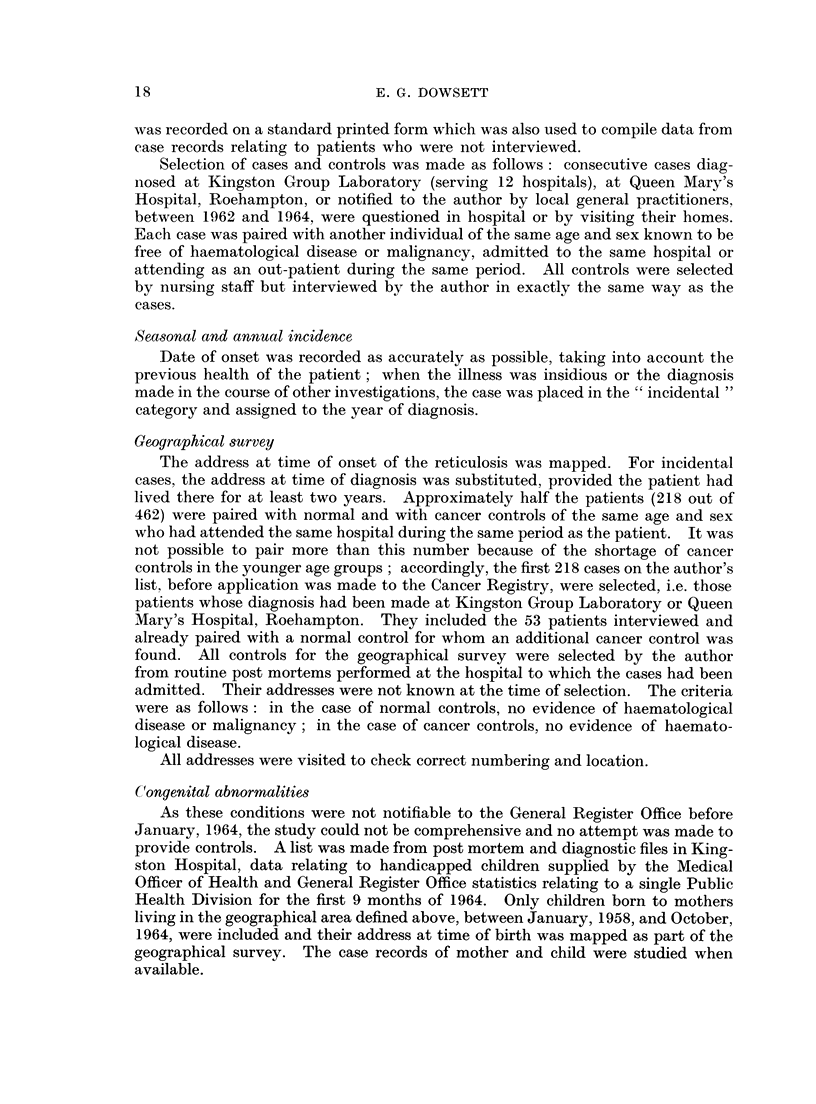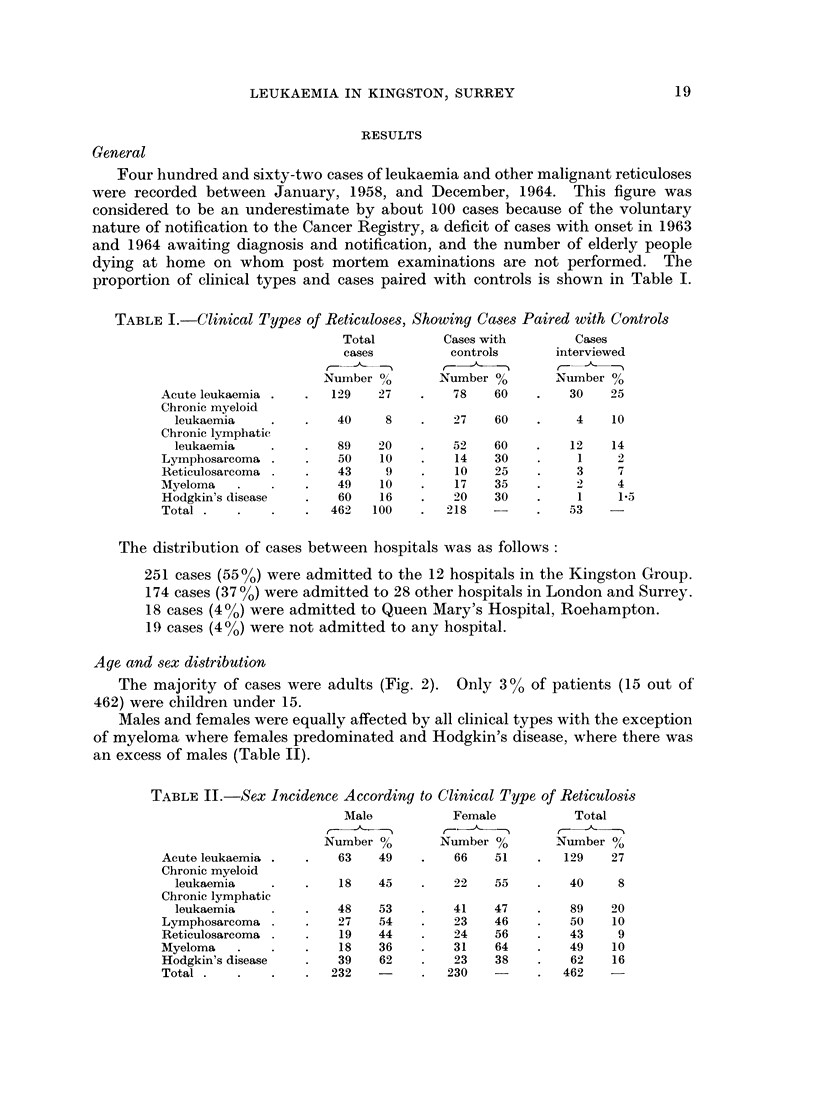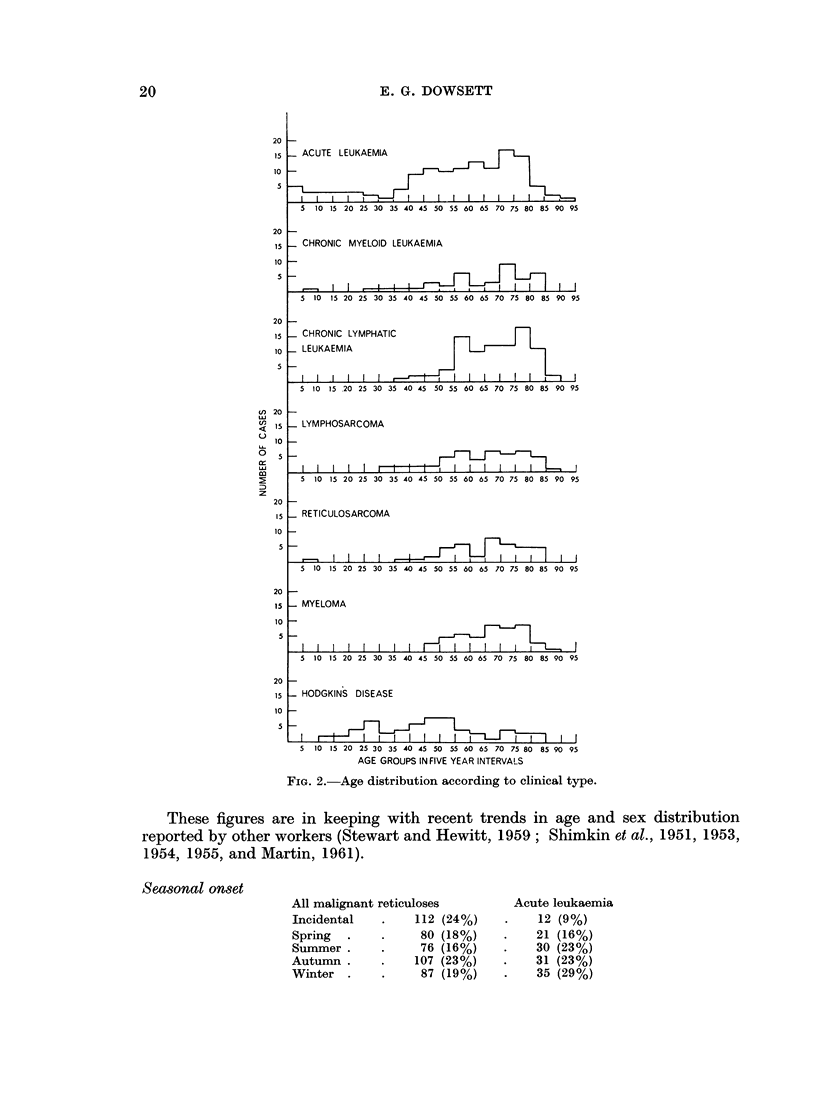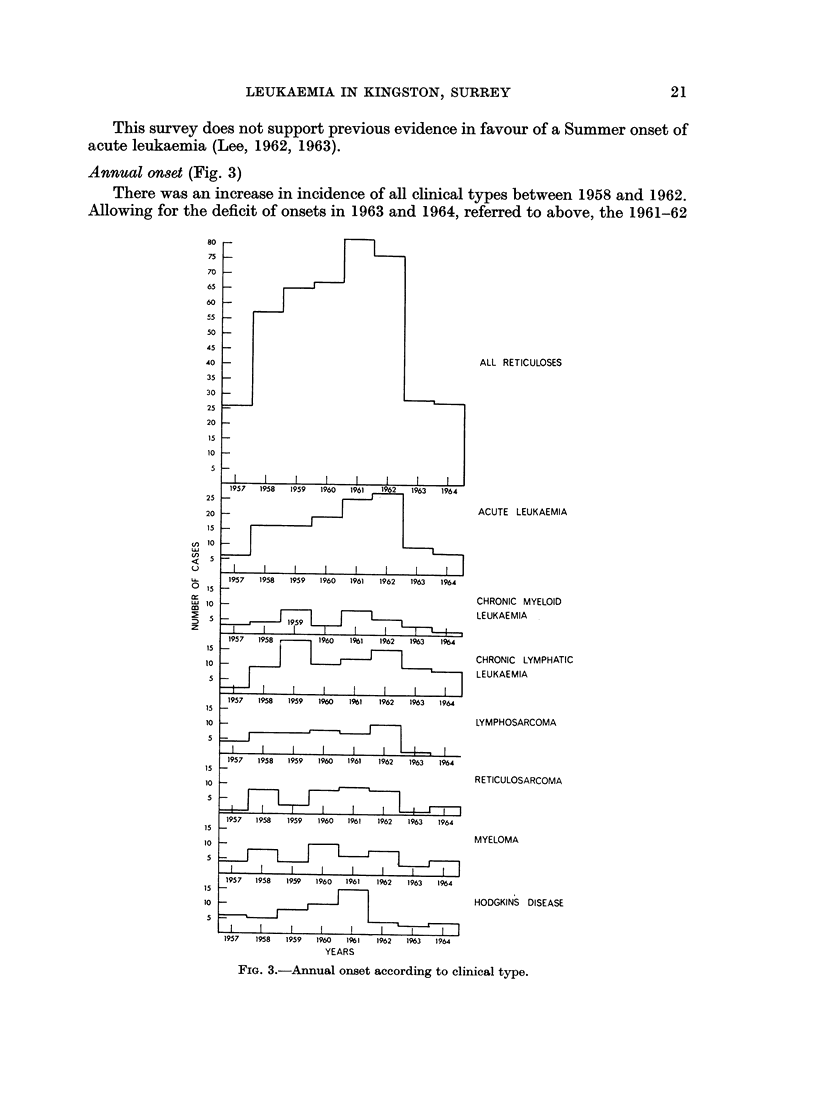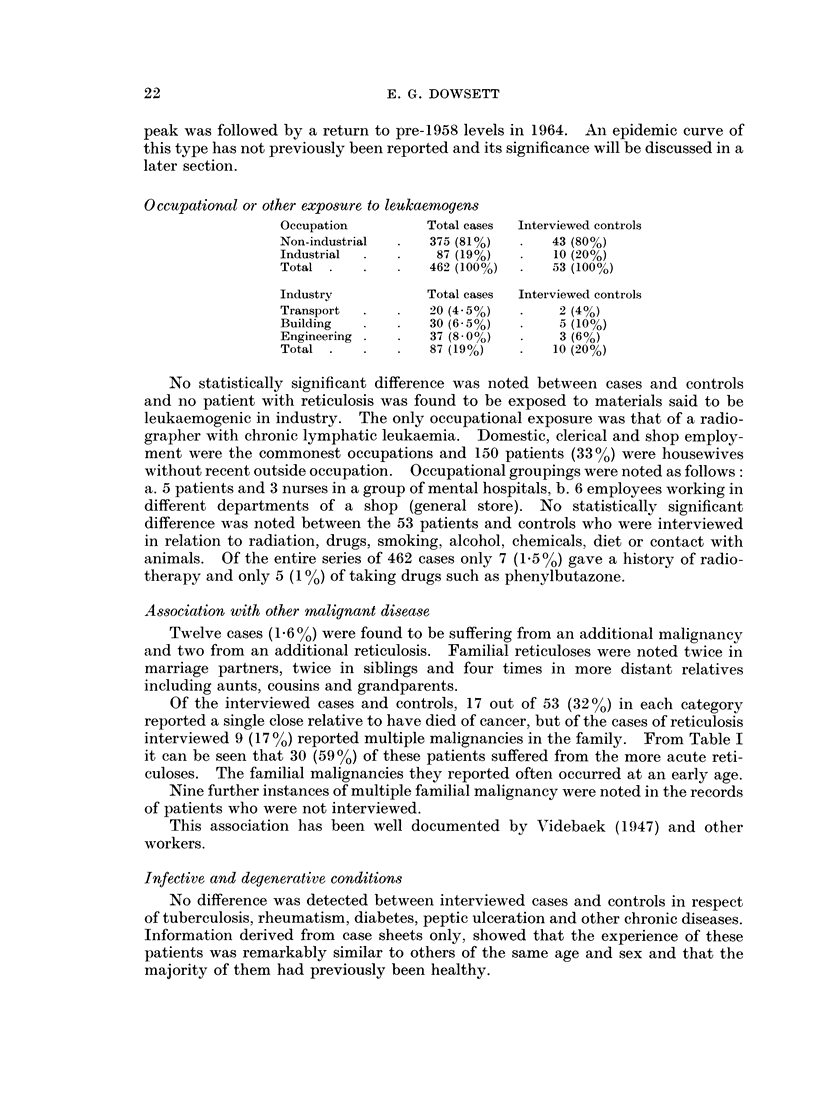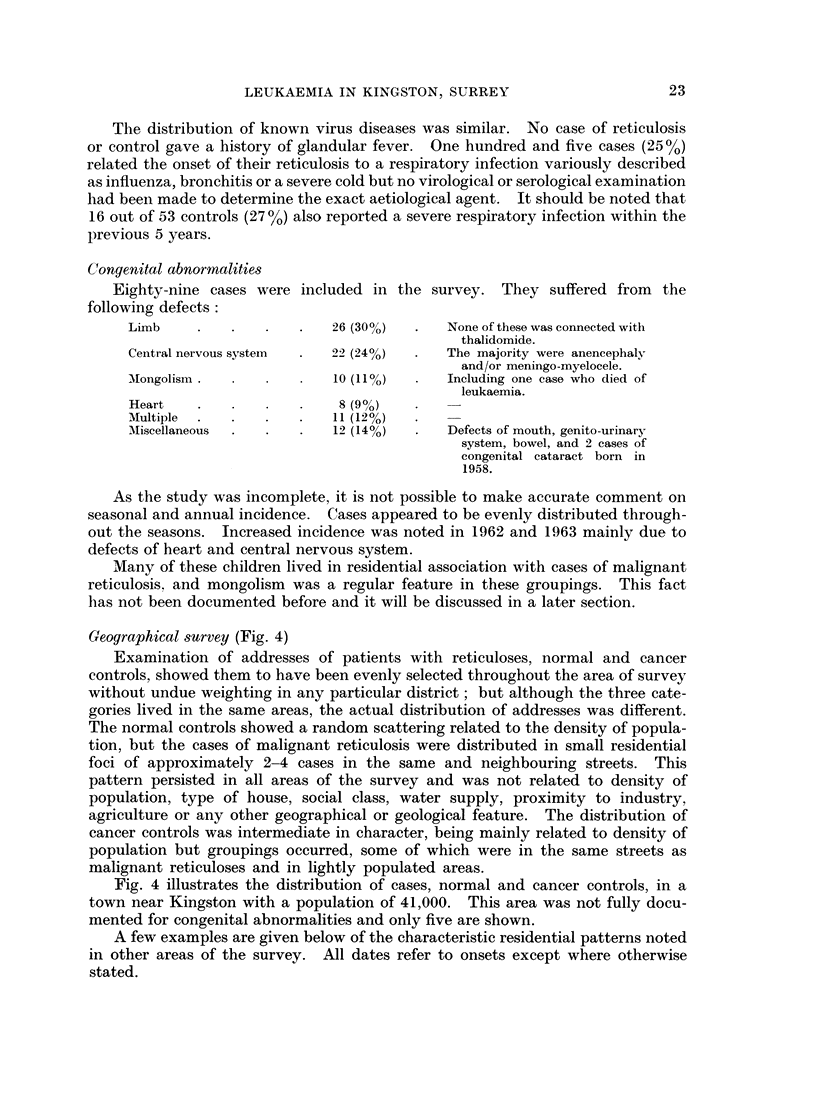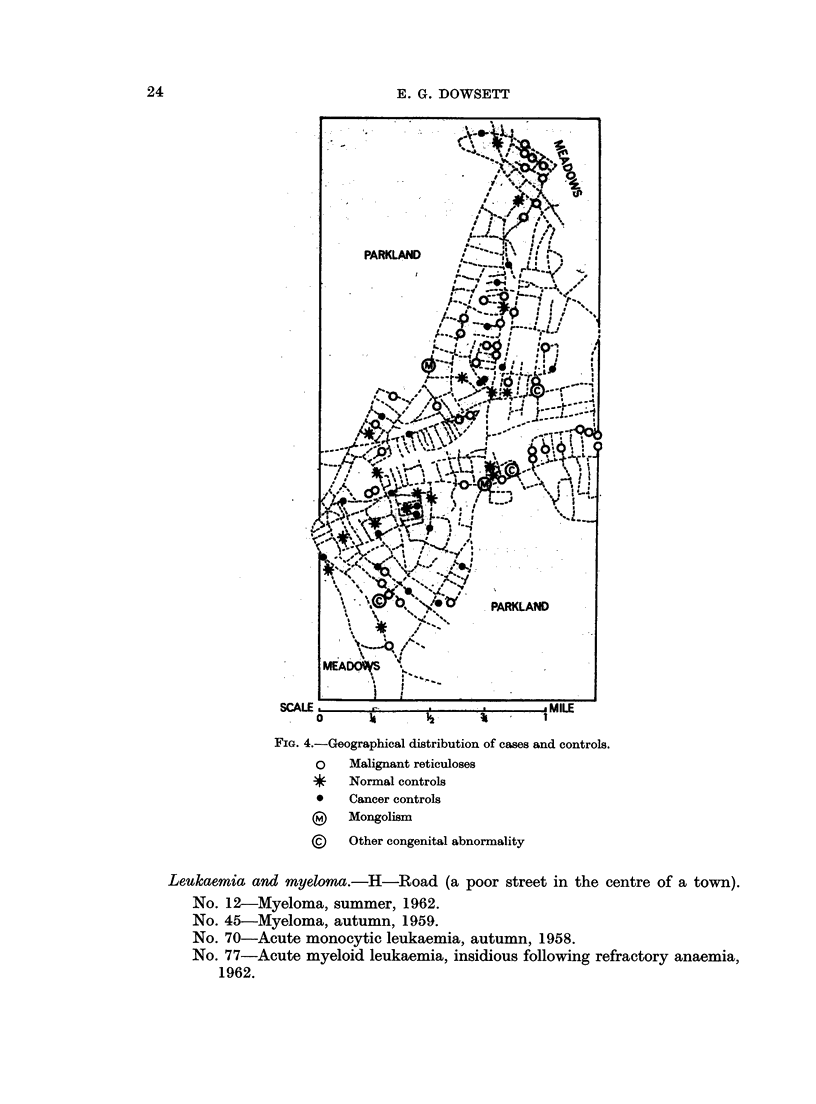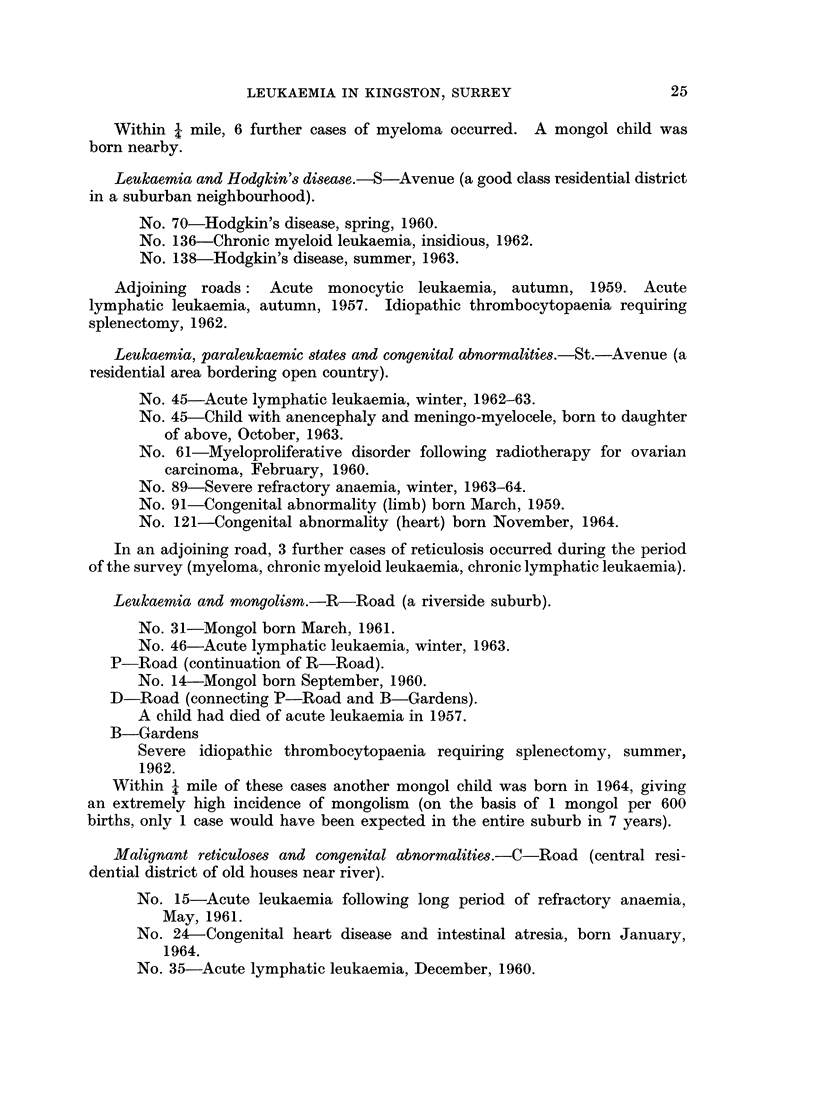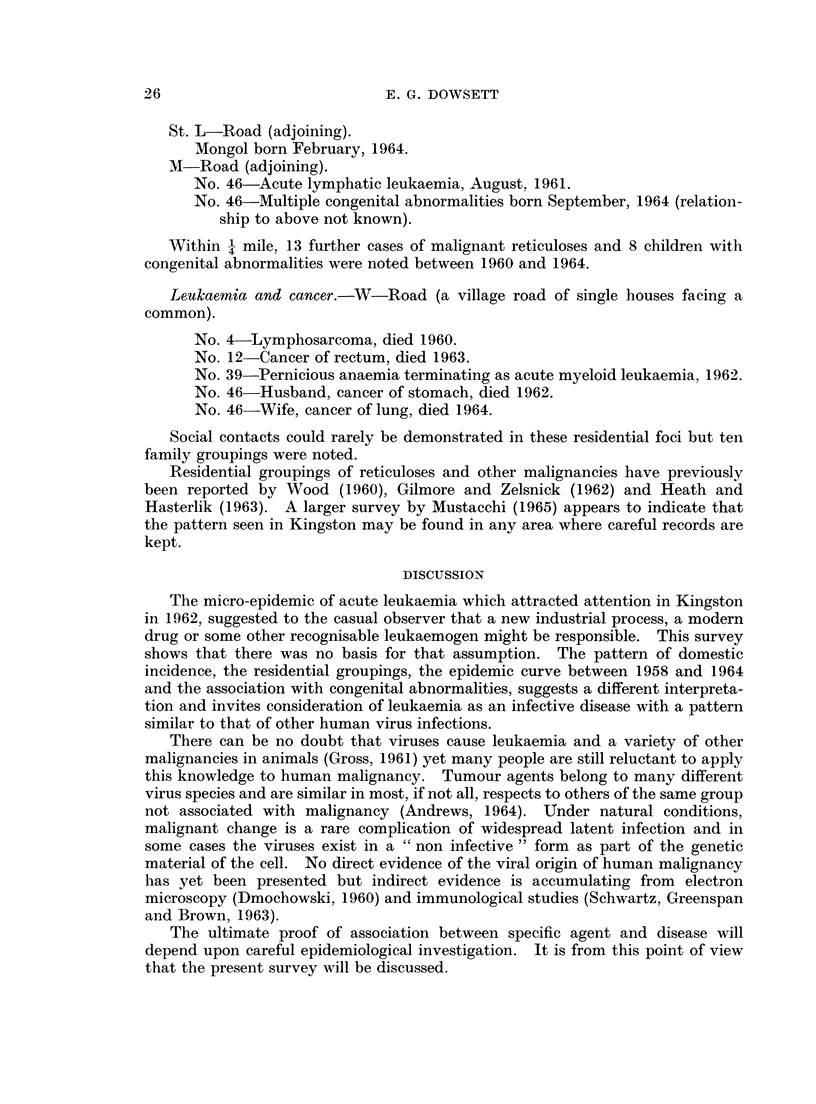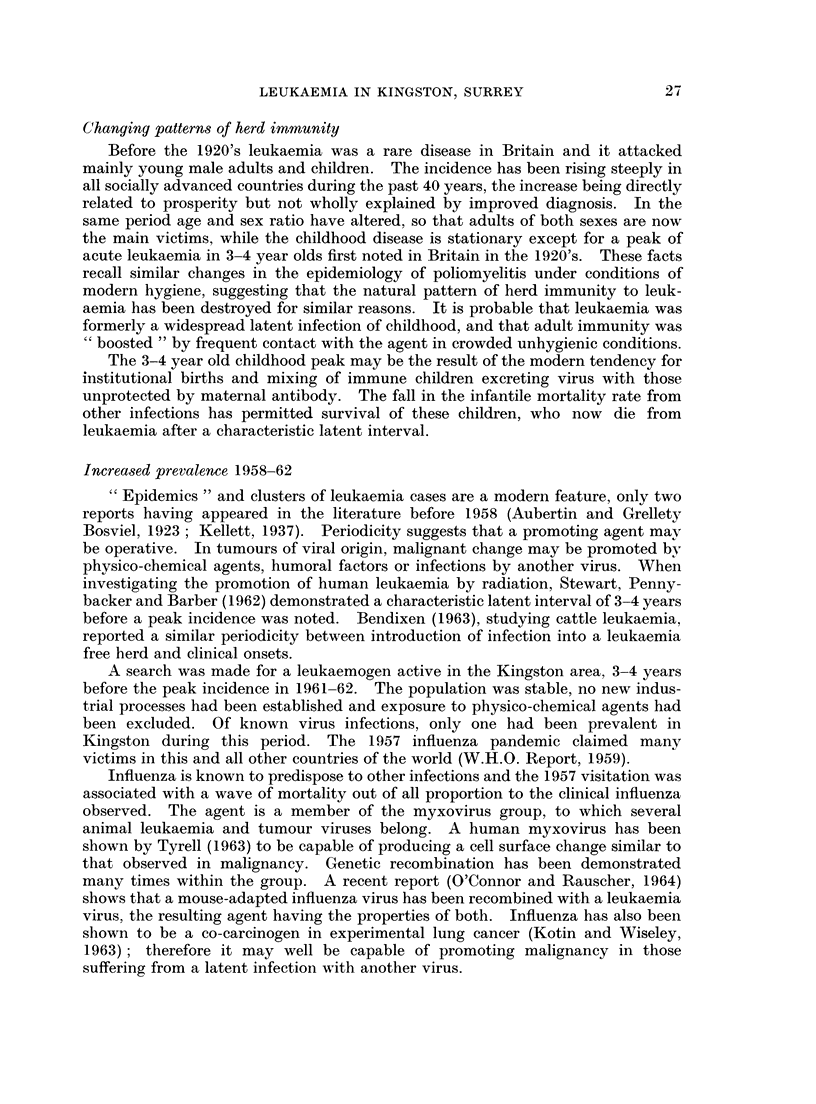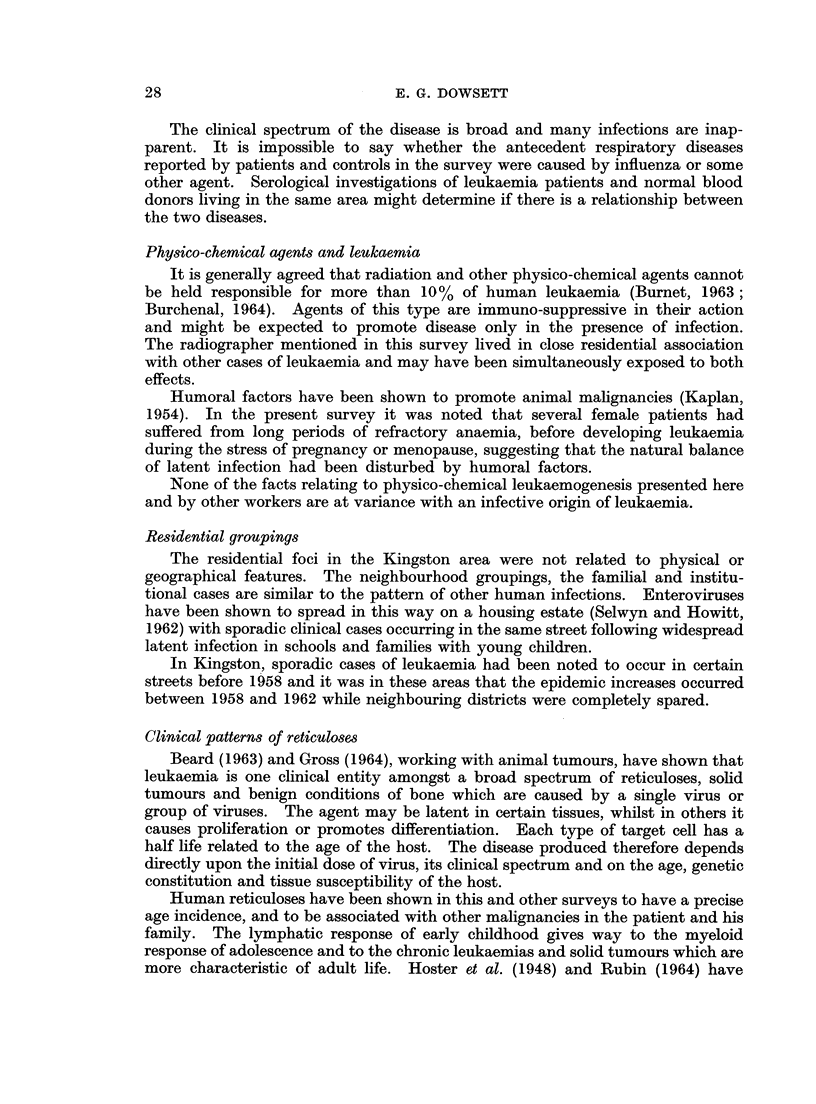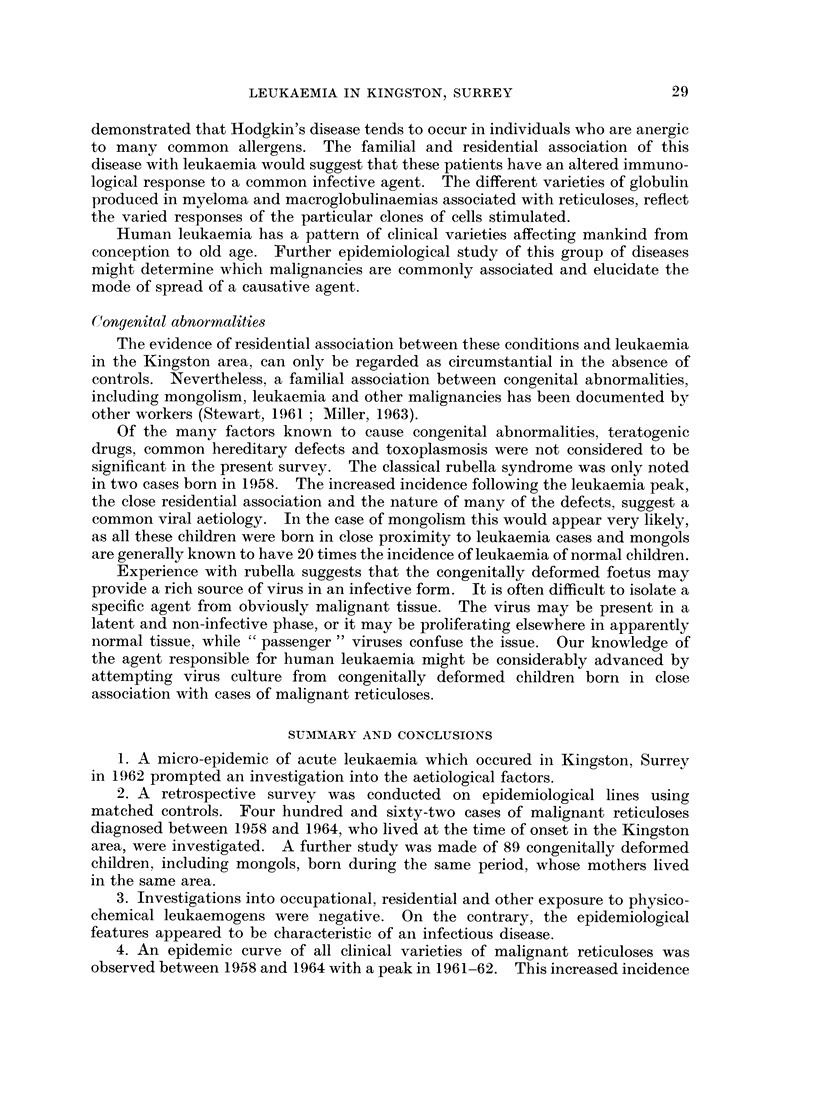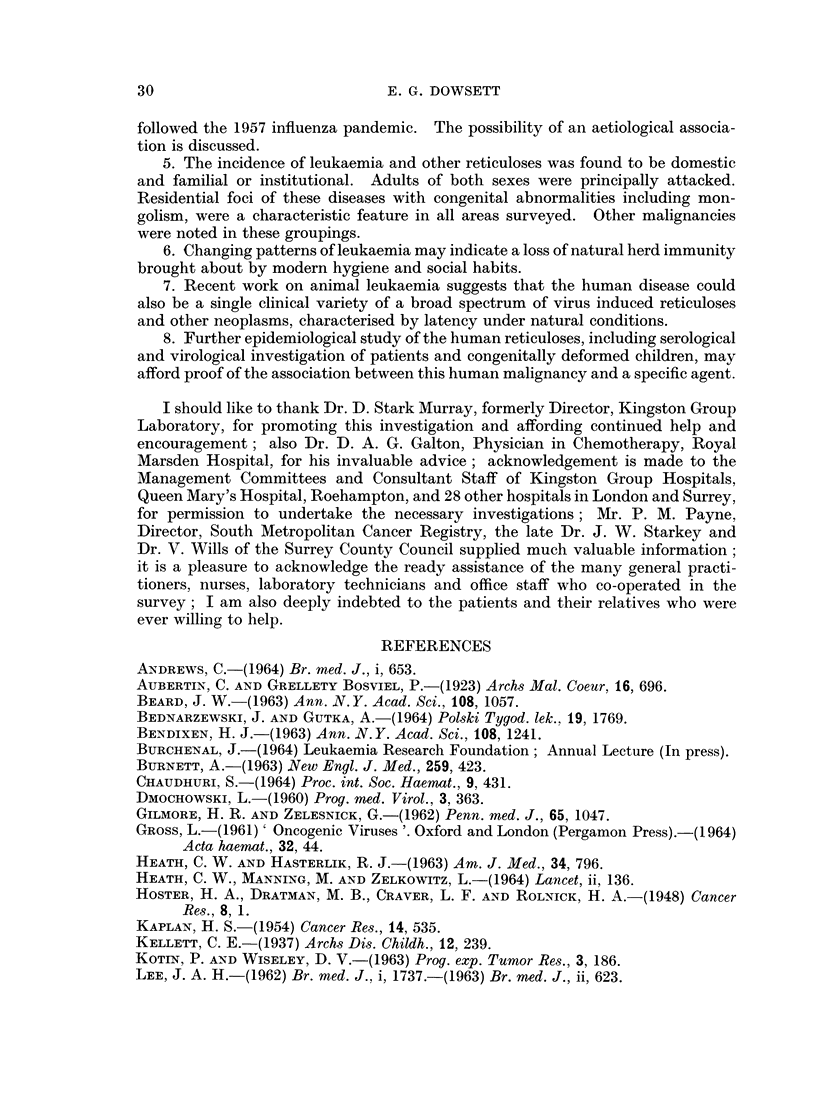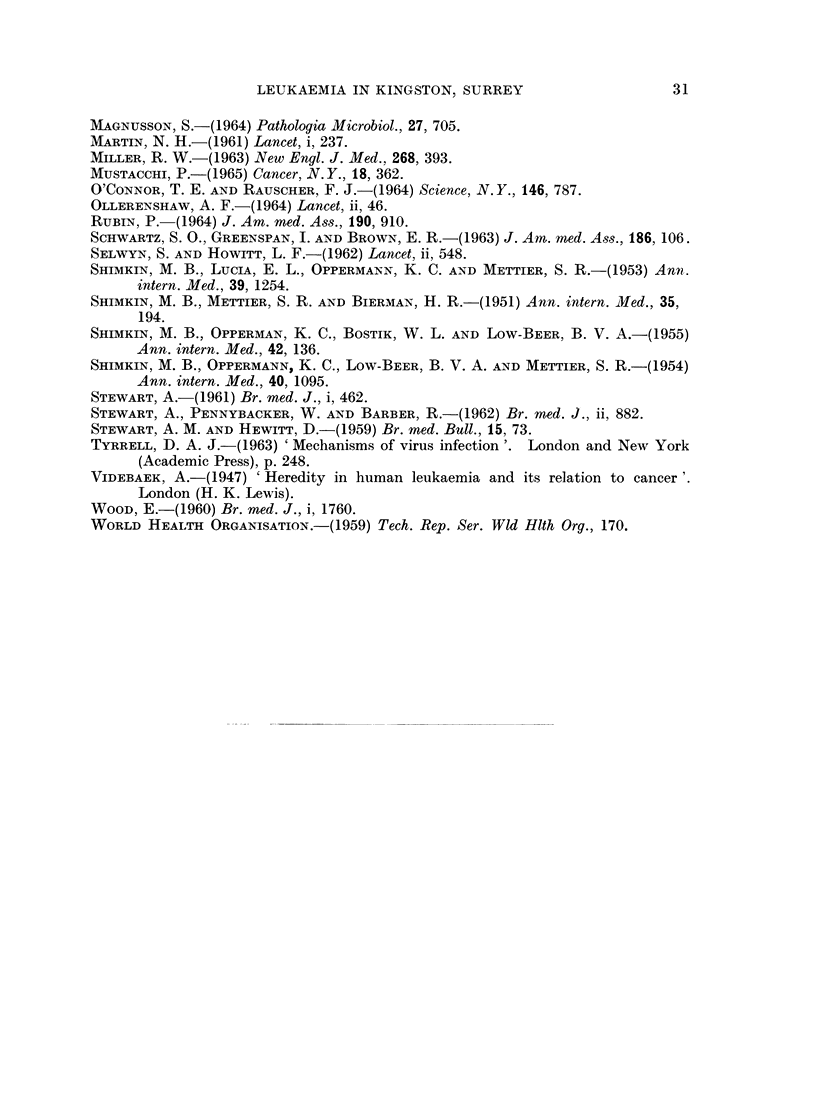# Leukaemia in Kingston, surrey, 1958-64: an epidemiological study.

**DOI:** 10.1038/bjc.1966.3

**Published:** 1966-03

**Authors:** E. G. Dowsett


### LEUKAEMIA IN KINGSTON, SURREY, 1958–64: AN EPIDEMIOLOGICAL STUDY

E. G. DOWSETT*

*From the Department of Pathology, Queen Mary's Hospital, Roehampton, London, S.W.15*

Received for publication November 11, 1965

DURING the first 6 months of 1962 14 cases of acute leukaemia were admitted to a General Hospital in Kingston, Surrey. In the same period, an increase in the incidence of other malignant reticuloses was noted. This micro-epidemic appeared to merit further investigation and the findings form the basis of this report. An increased incidence of leukaemia had been noted in other parts of Britain and of the world during this period, e.g. Lancashire (Ollerenshaw, 1964), South East England generally (P. M. Payne, personal communication), Poland (Bednarzewski and Gutka, 1964), India (Chaudhuri, 1964), Iceland (Magnusson, 1964), Niles, Illinois, U.S.A. (Heath and Hasterlik, 1963).

In the following year, congenital abnormalities appeared to be unusually prevalent. A supplementary investigation was made into the relationship between these events. A possible association between leukaemia outbreaks and congenital abnormalities was also reported from the U.S.A. (Heath, Manning and Zelkowitz, 1964).

The present study was undertaken with the intention of examining all known factors of aetiological significance. A comprehensive survey of this kind has not been presented before.

### METHODS

A retrospective survey, using matched controls, was made of all patients diagnosed as having leukaemia or other malignant reticuloses between 1958 and 1964 who lived at the time of onset of their illness in a defined geographical area.

### *Area of survey* (Fig. 1)

This was based on the catchment area of Kingston Group Laboratory, comprising 10 square miles in North Surrey, bordering the South-West London Suburbs. The geological and geographical features were diverse and the area included industrial, suburban residential and rural districts. The population of 450,000 was stable, prosperous, 95% British and consisted mainly of long-standing residents living in single houses. Aircraft manufacture and light engineering were the main local industries and some processes involved contact with materials said to be leukaemogenic. The area was exceptionally well supplied with medical and laboratory facilities and appeared to constitute an ideal background for a survey into leukaemogenesis.

*Present address :—Department of Bacteriology, St. George's Hospital, London, S.W.1.

FIG. 1.—Geographical area of survey.

*Recording of cases*

The diseases included in the survey as malignant reticuloses were acute leukaemia, chronic myeloid leukaemia, chronic lymphatic leukaemia, lymphosarcoma, reticulosarcoma, myeloma and Hodgkin's disease.

Information was obtained from laboratory records, post mortem and hospital diagnostic files. Details relating to patients treated at home or in hospitals outside the area were obtained from the local Cancer Registry where a record is kept of all death certificates and of cases notified by hospitals and general practitioners in South-East England. Permission was obtained from 41 hospitals in London and Surrey to make a personal examination of documents relating to these patients and from general practitioners in the case of those treated at home. Many relatives kindly assisted in these enquiries.

*Exposure of leukaemogens and other factors of aetiological significance*

A 10% sample (53 patients) together with their paired normal controls were questioned personally. The following details were particularly sought: place of birth, address at onset of illness, length of residence, previous addresses, age at onset, marital status, health of family or cause of death, previous illnesses including virus infections, date and mode of onset of reticulosis; occupation(s), exposure to radiation, drugs, chemicals, alcohol, smoking; any other factor of aetiological significance including diet and contact with animals. The information was recorded on a standard printed form which was also used to compile data from case records relating to patients who were not interviewed.

Selection of cases and controls was made as follows : consecutive cases diagnosed at Kingston Group Laboratory (serving 12 hospitals), at Queen Mary's Hospital, Roehampton, or notified to the author by local general practitioners, between 1962 and 1964, were questioned in hospital or by visiting their homes. Each case was paired with another individual of the same age and sex known to be free of haematological disease or malignancy, admitted to the same hospital or attending as an out-patient during the same period. All controls were selected by nursing staff but interviewed by the author in exactly the same way as the cases.

*Seasonal and annual incidence*

Date of onset was recorded as accurately as possible, taking into account the previous health of the patient ; when the illness was insidious or the diagnosis made in the course of other investigations, the case was placed in the " incidental " category and assigned to the year of diagnosis.

*Geographical survey*

The address at time of onset of the reticulosis was mapped. For incidental cases, the address at time of diagnosis was substituted, provided the patient had lived there for at least two years. Approximately half the patients (218 out of 462) were paired with normal and with cancer controls of the same age and sex who had attended the same hospital during the same period as the patient. It was not possible to pair more than this number because of the shortage of cancer controls in the younger age groups ; accordingly, the first 218 cases on the author's list, before application was made to the Cancer Registry, were selected, i.e. those patients whose diagnosis had been made at Kingston Group Laboratory or Queen Mary's Hospital, Roehampton. They included the 53 patients interviewed and already paired with a normal control for whom an additional cancer control was found. All controls for the geographical survey were selected by the author from routine post mortems performed at the hospital to which the cases had been admitted. Their addresses were not known at the time of selection. The criteria were as follows : in the case of normal controls, no evidence of haematological disease or malignancy ; in the case of cancer controls, no evidence of haematological disease.

All addresses were visited to check correct numbering and location.

*Congenital abnormalities*

As these conditions were not notifiable to the General Register Office before January, 1964, the study could not be comprehensive and no attempt was made to provide controls. A list was made from post mortem and diagnostic files in Kingston Hospital, data relating to handicapped children supplied by the Medical Officer of Health and General Register Office statistics relating to a single Public Health Division for the first 9 months of 1964. Only children born to mothers living in the geographical area defined above, between January, 1958, and October, 1964, were included and their address at time of birth was mapped as part of the geographical survey. The case records of mother and child were studied when available.

### RESULTS

*General*

Four hundred and sixty-two cases of leukaemia and other malignant reticuloses were recorded between January, 1958, and December, 1964. This figure was considered to be an underestimate by about 100 cases because of the voluntary nature of notification to the Cancer Registry, a deficit of cases with onset in 1963 and 1964 awaiting diagnosis and notification, and the number of elderly people dying at home on whom post mortem examinations are not performed. The proportion of clinical types and cases paired with controls is shown in Table I.

TABLE I.—*Clinical Types of Reticuloses, Showing Cases Paired with Controls*

| | Total cases | | Cases with controls | | Cases interviewed | |
|---|---|---|---|---|---|---|
| | Number | % | Number | % | Number | % |
| Acute leukaemia | 129 | 27 | 78 | 60 | 30 | 25 |
| Chronic myeloid leukaemia | 40 | 8 | 27 | 60 | 4 | 10 |
| Chronic lymphatic leukaemia | 89 | 20 | 52 | 60 | 12 | 14 |
| Lymphosarcoma | 50 | 10 | 14 | 30 | 1 | 2 |
| Reticulosarcoma | 43 | 9 | 10 | 25 | 3 | 7 |
| Myeloma | 49 | 10 | 17 | 35 | 2 | 4 |
| Hodgkin's disease | 60 | 16 | 20 | 30 | 1 | 1·5 |
| Total | 462 | 100 | 218 | — | 53 | — |

The distribution of cases between hospitals was as follows :

251 cases (55%) were admitted to the 12 hospitals in the Kingston Group.
174 cases (37%) were admitted to 28 other hospitals in London and Surrey.
18 cases (4%) were admitted to Queen Mary's Hospital, Roehampton.
19 cases (4%) were not admitted to any hospital.

*Age and sex distribution*

The majority of cases were adults (Fig. 2). Only 3% of patients (15 out of 462) were children under 15.

Males and females were equally affected by all clinical types with the exception of myeloma where females predominated and Hodgkin's disease, where there was an excess of males (Table II).

TABLE II.—*Sex Incidence According to Clinical Type of Reticulosis*

| | Male | | Female | | Total | |
|---|---|---|---|---|---|---|
| | Number | % | Number | % | Number | % |
| Acute leukaemia | 63 | 49 | 66 | 51 | 129 | 27 |
| Chronic myeloid leukaemia | 18 | 45 | 22 | 55 | 40 | 8 |
| Chronic lymphatic leukaemia | 48 | 53 | 41 | 47 | 89 | 20 |
| Lymphosarcoma | 27 | 54 | 23 | 46 | 50 | 10 |
| Reticulosarcoma | 19 | 44 | 24 | 56 | 43 | 9 |
| Myeloma | 18 | 36 | 31 | 64 | 49 | 10 |
| Hodgkin's disease | 39 | 62 | 23 | 38 | 62 | 16 |
| Total | 232 | — | 230 | — | 462 | — |

FIG. 2.—Age distribution according to clinical type.

These figures are in keeping with recent trends in age and sex distribution reported by other workers (Stewart and Hewitt, 1959 ; Shimkin *et al.*, 1951, 1953, 1954, 1955, and Martin, 1961).

*Seasonal onset*

| | All malignant reticuloses | Acute leukaemia |
|---|---|---|
| Incidental | 112 (24%) | 12 (9%) |
| Spring | 80 (18%) | 21 (16%) |
| Summer | 76 (16%) | 30 (23%) |
| Autumn | 107 (23%) | 31 (23%) |
| Winter | 87 (19%) | 35 (29%) |

This survey does not support previous evidence in favour of a Summer onset of acute leukaemia (Lee, 1962, 1963).

*Annual onset* (Fig. 3)

There was an increase in incidence of all clinical types between 1958 and 1962. Allowing for the deficit of onsets in 1963 and 1964, referred to above, the 1961–62

FIG. 3.—Annual onset according to clinical type.

peak was followed by a return to pre-1958 levels in 1964. An epidemic curve of this type has not previously been reported and its significance will be discussed in a later section.

*Occupational or other exposure to leukaemogens*

| Occupation | Total cases | Interviewed controls |
|---|---|---|
| Non-industrial | 375 (81%) | 43 (80%) |
| Industrial | 87 (19%) | 10 (20%) |
| Total | 462 (100%) | 53 (100%) |

| Industry | Total cases | Interviewed controls |
|---|---|---|
| Transport | 20 (4·5%) | 2 (4%) |
| Building | 30 (6·5%) | 5 (10%) |
| Engineering | 37 (8·0%) | 3 (6%) |
| Total | 87 (19%) | 10 (20%) |

No statistically significant difference was noted between cases and controls and no patient with reticulosis was found to be exposed to materials said to be leukaemogenic in industry. The only occupational exposure was that of a radiographer with chronic lymphatic leukaemia. Domestic, clerical and shop employment were the commonest occupations and 150 patients (33%) were housewives without recent outside occupation. Occupational groupings were noted as follows: a. 5 patients and 3 nurses in a group of mental hospitals, b. 6 employees working in different departments of a shop (general store). No statistically significant difference was noted between the 53 patients and controls who were interviewed in relation to radiation, drugs, smoking, alcohol, chemicals, diet or contact with animals. Of the entire series of 462 cases only 7 (1·5%) gave a history of radiotherapy and only 5 (1%) of taking drugs such as phenylbutazone.

*Association with other malignant disease*

Twelve cases (1·6%) were found to be suffering from an additional malignancy and two from an additional reticulosis. Familial reticuloses were noted twice in marriage partners, twice in siblings and four times in more distant relatives including aunts, cousins and grandparents.

Of the interviewed cases and controls, 17 out of 53 (32%) in each category reported a single close relative to have died of cancer, but of the cases of reticulosis interviewed 9 (17%) reported multiple malignancies in the family. From Table I it can be seen that 30 (59%) of these patients suffered from the more acute reticuloses. The familial malignancies they reported often occurred at an early age.

Nine further instances of multiple familial malignancy were noted in the records of patients who were not interviewed.

This association has been well documented by Videbaek (1947) and other workers.

*Infective and degenerative conditions*

No difference was detected between interviewed cases and controls in respect of tuberculosis, rheumatism, diabetes, peptic ulceration and other chronic diseases. Information derived from case sheets only, showed that the experience of these patients was remarkably similar to others of the same age and sex and that the majority of them had previously been healthy.

The distribution of known virus diseases was similar. No case of reticulosis or control gave a history of glandular fever. One hundred and five cases (25%) related the onset of their reticulosis to a respiratory infection variously described as influenza, bronchitis or a severe cold but no virological or serological examination had been made to determine the exact aetiological agent. It should be noted that 16 out of 53 controls (27%) also reported a severe respiratory infection within the previous 5 years.

*Congenital abnormalities*

Eighty-nine cases were included in the survey. They suffered from the following defects :

| | | |
|---|---|---|
| Limb | 26 (30%) | None of these was connected with thalidomide. |
| Central nervous system | 22 (24%) | The majority were anencephaly and/or meningo-myelocele. |
| Mongolism | 10 (11%) | Including one case who died of leukaemia. |
| Heart | 8 (9%) | — |
| Multiple | 11 (12%) | — |
| Miscellaneous | 12 (14%) | Defects of mouth, genito-urinary system, bowel, and 2 cases of congenital cataract born in 1958. |

As the study was incomplete, it is not possible to make accurate comment on seasonal and annual incidence. Cases appeared to be evenly distributed throughout the seasons. Increased incidence was noted in 1962 and 1963 mainly due to defects of heart and central nervous system.

Many of these children lived in residential association with cases of malignant reticulosis, and mongolism was a regular feature in these groupings. This fact has not been documented before and it will be discussed in a later section.

*Geographical survey* (Fig. 4)

Examination of addresses of patients with reticuloses, normal and cancer controls, showed them to have been evenly selected throughout the area of survey without undue weighting in any particular district ; but although the three categories lived in the same areas, the actual distribution of addresses was different. The normal controls showed a random scattering related to the density of population, but the cases of malignant reticulosis were distributed in small residential foci of approximately 2–4 cases in the same and neighbouring streets. This pattern persisted in all areas of the survey and was not related to density of population, type of house, social class, water supply, proximity to industry, agriculture or any other geographical or geological feature. The distribution of cancer controls was intermediate in character, being mainly related to density of population but groupings occurred, some of which were in the same streets as malignant reticuloses and in lightly populated areas.

Fig. 4 illustrates the distribution of cases, normal and cancer controls, in a town near Kingston with a population of 41,000. This area was not fully documented for congenital abnormalities and only five are shown.

A few examples are given below of the characteristic residential patterns noted in other areas of the survey. All dates refer to onsets except where otherwise stated.

**Fig. 4.—Geographical distribution of cases and controls.**

- O Malignant reticuloses
- ✳ Normal controls
- ● Cancer controls
- Ⓜ Mongolism
- © Other congenital abnormality

*Leukaemia and myeloma.*—H—Road (a poor street in the centre of a town).

No. 12—Myeloma, summer, 1962.
No. 45—Myeloma, autumn, 1959.
No. 70—Acute monocytic leukaemia, autumn, 1958.
No. 77—Acute myeloid leukaemia, insidious following refractory anaemia, 1962.

Within $\frac{1}{4}$ mile, 6 further cases of myeloma occurred. A mongol child was born nearby.

*Leukaemia and Hodgkin's disease.*—S—Avenue (a good class residential district in a suburban neighbourhood).

No. 70—Hodgkin's disease, spring, 1960.
No. 136—Chronic myeloid leukaemia, insidious, 1962.
No. 138—Hodgkin's disease, summer, 1963.

Adjoining roads: Acute monocytic leukaemia, autumn, 1959. Acute lymphatic leukaemia, autumn, 1957. Idiopathic thrombocytopaenia requiring splenectomy, 1962.

*Leukaemia, paraleukaemic states and congenital abnormalities.*—St.—Avenue (a residential area bordering open country).

No. 45—Acute lymphatic leukaemia, winter, 1962–63.
No. 45—Child with anencephaly and meningo-myelocele, born to daughter of above, October, 1963.
No. 61—Myeloproliferative disorder following radiotherapy for ovarian carcinoma, February, 1960.
No. 89—Severe refractory anaemia, winter, 1963–64.
No. 91—Congenital abnormality (limb) born March, 1959.
No. 121—Congenital abnormality (heart) born November, 1964.

In an adjoining road, 3 further cases of reticulosis occurred during the period of the survey (myeloma, chronic myeloid leukaemia, chronic lymphatic leukaemia).

*Leukaemia and mongolism.*—R—Road (a riverside suburb).

No. 31—Mongol born March, 1961.
No. 46—Acute lymphatic leukaemia, winter, 1963.

P—Road (continuation of R—Road).

No. 14—Mongol born September, 1960.

D—Road (connecting P—Road and B—Gardens).

A child had died of acute leukaemia in 1957.

B—Gardens

Severe idiopathic thrombocytopaenia requiring splenectomy, summer, 1962.

Within $\frac{1}{4}$ mile of these cases another mongol child was born in 1964, giving an extremely high incidence of mongolism (on the basis of 1 mongol per 600 births, only 1 case would have been expected in the entire suburb in 7 years).

*Malignant reticuloses and congenital abnormalities.*—C—Road (central residential district of old houses near river).

No. 15—Acute leukaemia following long period of refractory anaemia, May, 1961.
No. 24—Congenital heart disease and intestinal atresia, born January, 1964.
No. 35—Acute lymphatic leukaemia, December, 1960.

St. L—Road (adjoining).
Mongol born February, 1964.
M—Road (adjoining).
No. 46—Acute lymphatic leukaemia, August, 1961.
No. 46—Multiple congenital abnormalities born September, 1964 (relationship to above not known).

Within $\frac{1}{4}$ mile, 13 further cases of malignant reticuloses and 8 children with congenital abnormalities were noted between 1960 and 1964.

*Leukaemia and cancer.*—W—Road (a village road of single houses facing a common).

No. 4—Lymphosarcoma, died 1960.
No. 12—Cancer of rectum, died 1963.
No. 39—Pernicious anaemia terminating as acute myeloid leukaemia, 1962.
No. 46—Husband, cancer of stomach, died 1962.
No. 46—Wife, cancer of lung, died 1964.

Social contacts could rarely be demonstrated in these residential foci but ten family groupings were noted.

Residential groupings of reticuloses and other malignancies have previously been reported by Wood (1960), Gilmore and Zelsnick (1962) and Heath and Hasterlik (1963). A larger survey by Mustacchi (1965) appears to indicate that the pattern seen in Kingston may be found in any area where careful records are kept.

### DISCUSSION

The micro-epidemic of acute leukaemia which attracted attention in Kingston in 1962, suggested to the casual observer that a new industrial process, a modern drug or some other recognisable leukaemogen might be responsible. This survey shows that there was no basis for that assumption. The pattern of domestic incidence, the residential groupings, the epidemic curve between 1958 and 1964 and the association with congenital abnormalities, suggests a different interpretation and invites consideration of leukaemia as an infective disease with a pattern similar to that of other human virus infections.

There can be no doubt that viruses cause leukaemia and a variety of other malignancies in animals (Gross, 1961) yet many people are still reluctant to apply this knowledge to human malignancy. Tumour agents belong to many different virus species and are similar in most, if not all, respects to others of the same group not associated with malignancy (Andrews, 1964). Under natural conditions, malignant change is a rare complication of widespread latent infection and in some cases the viruses exist in a " non infective " form as part of the genetic material of the cell. No direct evidence of the viral origin of human malignancy has yet been presented but indirect evidence is accumulating from electron microscopy (Dmochowski, 1960) and immunological studies (Schwartz, Greenspan and Brown, 1963).

The ultimate proof of association between specific agent and disease will depend upon careful epidemiological investigation. It is from this point of view that the present survey will be discussed.

*Changing patterns of herd immunity*

Before the 1920's leukaemia was a rare disease in Britain and it attacked mainly young male adults and children. The incidence has been rising steeply in all socially advanced countries during the past 40 years, the increase being directly related to prosperity but not wholly explained by improved diagnosis. In the same period age and sex ratio have altered, so that adults of both sexes are now the main victims, while the childhood disease is stationary except for a peak of acute leukaemia in 3–4 year olds first noted in Britain in the 1920's. These facts recall similar changes in the epidemiology of poliomyelitis under conditions of modern hygiene, suggesting that the natural pattern of herd immunity to leukaemia has been destroyed for similar reasons. It is probable that leukaemia was formerly a widespread latent infection of childhood, and that adult immunity was " boosted " by frequent contact with the agent in crowded unhygienic conditions.

The 3–4 year old childhood peak may be the result of the modern tendency for institutional births and mixing of immune children excreting virus with those unprotected by maternal antibody. The fall in the infantile mortality rate from other infections has permitted survival of these children, who now die from leukaemia after a characteristic latent interval.

*Increased prevalence* 1958–62

" Epidemics " and clusters of leukaemia cases are a modern feature, only two reports having appeared in the literature before 1958 (Aubertin and Grellety Bosviel, 1923 ; Kellett, 1937). Periodicity suggests that a promoting agent may be operative. In tumours of viral origin, malignant change may be promoted by physico-chemical agents, humoral factors or infections by another virus. When investigating the promotion of human leukaemia by radiation, Stewart, Pennybacker and Barber (1962) demonstrated a characteristic latent interval of 3–4 years before a peak incidence was noted. Bendixen (1963), studying cattle leukaemia, reported a similar periodicity between introduction of infection into a leukaemia free herd and clinical onsets.

A search was made for a leukaemogen active in the Kingston area, 3–4 years before the peak incidence in 1961–62. The population was stable, no new industrial processes had been established and exposure to physico-chemical agents had been excluded. Of known virus infections, only one had been prevalent in Kingston during this period. The 1957 influenza pandemic claimed many victims in this and all other countries of the world (W.H.O. Report, 1959).

Influenza is known to predispose to other infections and the 1957 visitation was associated with a wave of mortality out of all proportion to the clinical influenza observed. The agent is a member of the myxovirus group, to which several animal leukaemia and tumour viruses belong. A human myxovirus has been shown by Tyrell (1963) to be capable of producing a cell surface change similar to that observed in malignancy. Genetic recombination has been demonstrated many times within the group. A recent report (O'Connor and Rauscher, 1964) shows that a mouse-adapted influenza virus has been recombined with a leukaemia virus, the resulting agent having the properties of both. Influenza has also been shown to be a co-carcinogen in experimental lung cancer (Kotin and Wiseley, 1963) ; therefore it may well be capable of promoting malignancy in those suffering from a latent infection with another virus.

The clinical spectrum of the disease is broad and many infections are inapparent. It is impossible to say whether the antecedent respiratory diseases reported by patients and controls in the survey were caused by influenza or some other agent. Serological investigations of leukaemia patients and normal blood donors living in the same area might determine if there is a relationship between the two diseases.

*Physico-chemical agents and leukaemia*

It is generally agreed that radiation and other physico-chemical agents cannot be held responsible for more than 10% of human leukaemia (Burnet, 1963; Burchenal, 1964). Agents of this type are immuno-suppressive in their action and might be expected to promote disease only in the presence of infection. The radiographer mentioned in this survey lived in close residential association with other cases of leukaemia and may have been simultaneously exposed to both effects.

Humoral factors have been shown to promote animal malignancies (Kaplan, 1954). In the present survey it was noted that several female patients had suffered from long periods of refractory anaemia, before developing leukaemia during the stress of pregnancy or menopause, suggesting that the natural balance of latent infection had been disturbed by humoral factors.

None of the facts relating to physico-chemical leukaemogenesis presented here and by other workers are at variance with an infective origin of leukaemia.

*Residential groupings*

The residential foci in the Kingston area were not related to physical or geographical features. The neighbourhood groupings, the familial and institutional cases are similar to the pattern of other human infections. Enteroviruses have been shown to spread in this way on a housing estate (Selwyn and Howitt, 1962) with sporadic clinical cases occurring in the same street following widespread latent infection in schools and families with young children.

In Kingston, sporadic cases of leukaemia had been noted to occur in certain streets before 1958 and it was in these areas that the epidemic increases occurred between 1958 and 1962 while neighbouring districts were completely spared.

*Clinical patterns of reticuloses*

Beard (1963) and Gross (1964), working with animal tumours, have shown that leukaemia is one clinical entity amongst a broad spectrum of reticuloses, solid tumours and benign conditions of bone which are caused by a single virus or group of viruses. The agent may be latent in certain tissues, whilst in others it causes proliferation or promotes differentiation. Each type of target cell has a half life related to the age of the host. The disease produced therefore depends directly upon the initial dose of virus, its clinical spectrum and on the age, genetic constitution and tissue susceptibility of the host.

Human reticuloses have been shown in this and other surveys to have a precise age incidence, and to be associated with other malignancies in the patient and his family. The lymphatic response of early childhood gives way to the myeloid response of adolescence and to the chronic leukaemias and solid tumours which are more characteristic of adult life. Hoster *et al.* (1948) and Rubin (1964) have demonstrated that Hodgkin's disease tends to occur in individuals who are anergic to many common allergens. The familial and residential association of this disease with leukaemia would suggest that these patients have an altered immunological response to a common infective agent. The different varieties of globulin produced in myeloma and macroglobulinaemias associated with reticuloses, reflect the varied responses of the particular clones of cells stimulated.

Human leukaemia has a pattern of clinical varieties affecting mankind from conception to old age. Further epidemiological study of this group of diseases might determine which malignancies are commonly associated and elucidate the mode of spread of a causative agent.

*Congenital abnormalities*

The evidence of residential association between these conditions and leukaemia in the Kingston area, can only be regarded as circumstantial in the absence of controls. Nevertheless, a familial association between congenital abnormalities, including mongolism, leukaemia and other malignancies has been documented by other workers (Stewart, 1961 ; Miller, 1963).

Of the many factors known to cause congenital abnormalities, teratogenic drugs, common hereditary defects and toxoplasmosis were not considered to be significant in the present survey. The classical rubella syndrome was only noted in two cases born in 1958. The increased incidence following the leukaemia peak, the close residential association and the nature of many of the defects, suggest a common viral aetiology. In the case of mongolism this would appear very likely, as all these children were born in close proximity to leukaemia cases and mongols are generally known to have 20 times the incidence of leukaemia of normal children.

Experience with rubella suggests that the congenitally deformed foetus may provide a rich source of virus in an infective form. It is often difficult to isolate a specific agent from obviously malignant tissue. The virus may be present in a latent and non-infective phase, or it may be proliferating elsewhere in apparently normal tissue, while " passenger " viruses confuse the issue. Our knowledge of the agent responsible for human leukaemia might be considerably advanced by attempting virus culture from congenitally deformed children born in close association with cases of malignant reticuloses.

### SUMMARY AND CONCLUSIONS

1. A micro-epidemic of acute leukaemia which occured in Kingston, Surrey in 1962 prompted an investigation into the aetiological factors.

2. A retrospective survey was conducted on epidemiological lines using matched controls. Four hundred and sixty-two cases of malignant reticuloses diagnosed between 1958 and 1964, who lived at the time of onset in the Kingston area, were investigated. A further study was made of 89 congenitally deformed children, including mongols, born during the same period, whose mothers lived in the same area.

3. Investigations into occupational, residential and other exposure to physicochemical leukaemogens were negative. On the contrary, the epidemiological features appeared to be characteristic of an infectious disease.

4. An epidemic curve of all clinical varieties of malignant reticuloses was observed between 1958 and 1964 with a peak in 1961–62. This increased incidence followed the 1957 influenza pandemic. The possibility of an aetiological association is discussed.

5. The incidence of leukaemia and other reticuloses was found to be domestic and familial or institutional. Adults of both sexes were principally attacked. Residential foci of these diseases with congenital abnormalities including mongolism, were a characteristic feature in all areas surveyed. Other malignancies were noted in these groupings.

6. Changing patterns of leukaemia may indicate a loss of natural herd immunity brought about by modern hygiene and social habits.

7. Recent work on animal leukaemia suggests that the human disease could also be a single clinical variety of a broad spectrum of virus induced reticuloses and other neoplasms, characterised by latency under natural conditions.

8. Further epidemiological study of the human reticuloses, including serological and virological investigation of patients and congenitally deformed children, may afford proof of the association between this human malignancy and a specific agent.

I should like to thank Dr. D. Stark Murray, formerly Director, Kingston Group Laboratory, for promoting this investigation and affording continued help and encouragement ; also Dr. D. A. G. Galton, Physician in Chemotherapy, Royal Marsden Hospital, for his invaluable advice ; acknowledgement is made to the Management Committees and Consultant Staff of Kingston Group Hospitals, Queen Mary's Hospital, Roehampton, and 28 other hospitals in London and Surrey, for permission to undertake the necessary investigations ; Mr. P. M. Payne, Director, South Metropolitan Cancer Registry, the late Dr. J. W. Starkey and Dr. V. Wills of the Surrey County Council supplied much valuable information ; it is a pleasure to acknowledge the ready assistance of the many general practitioners, nurses, laboratory technicians and office staff who co-operated in the survey ; I am also deeply indebted to the patients and their relatives who were ever willing to help.

### REFERENCES

ANDREWS, C.—(1964) *Br. med. J.*, i, 653.
AUBERTIN, C. AND GRELLETY BOSVIEL, P.—(1923) *Archs Mal. Coeur*, **16**, 696.
BEARD, J. W.—(1963) *Ann. N.Y. Acad. Sci.*, **108**, 1057.
BEDNARZEWSKI, J. AND GUTKA, A.—(1964) *Polski Tygod. lek.*, **19**, 1769.
BENDIXEN, H. J.—(1963) *Ann. N.Y. Acad. Sci.*, **108**, 1241.
BURCHENAL, J.—(1964) Leukaemia Research Foundation ; Annual Lecture (In press).
BURNETT, A.—(1963) *New Engl. J. Med.*, **259**, 423.
CHAUDHURI, S.—(1964) *Proc. int. Soc. Haemat.*, **9**, 431.
DMOCHOWSKI, L.—(1960) *Prog. med. Virol.*, **3**, 363.
GILMORE, H. R. AND ZELESNICK, G.—(1962) *Penn. med. J.*, **65**, 1047.
GROSS, L.—(1961) ' Oncogenic Viruses '. Oxford and London (Pergamon Press).—(1964) *Acta haemat.*, **32**, 44.
HEATH, C. W. AND HASTERLIK, R. J.—(1963) *Am. J. Med.*, **34**, 796.
HEATH, C. W., MANNING, M. AND ZELKOWITZ, L.—(1964) *Lancet*, ii, 136.
HOSTER, H. A., DRATMAN, M. B., CRAVER, L. F. AND ROLNICK, H. A.—(1948) *Cancer Res.*, **8**, 1.
KAPLAN, H. S.—(1954) *Cancer Res.*, **14**, 535.
KELLETT, C. E.—(1937) *Archs Dis. Childh.*, **12**, 239.
KOTIN, P. AND WISELEY, D. V.—(1963) *Prog. exp. Tumor Res.*, **3**, 186.
LEE, J. A. H.—(1962) *Br. med. J.*, i, 1737.—(1963) *Br. med. J.*, ii, 623.

Magnusson, S.—(1964) *Pathologia Microbiol.*, **27**, 705.
Martin, N. H.—(1961) *Lancet*, i, 237.
Miller, R. W.—(1963) *New Engl. J. Med.*, **268**, 393.
Mustacchi, P.—(1965) *Cancer, N.Y.*, **18**, 362.
O'Connor, T. E. and Rauscher, F. J.—(1964) *Science, N.Y.*, **146**, 787.
Ollerenshaw, A. F.—(1964) *Lancet*, ii, 46.
Rubin, P.—(1964) *J. Am. med. Ass.*, **190**, 910.
Schwartz, S. O., Greenspan, I. and Brown, E. R.—(1963) *J. Am. med. Ass.*, **186**, 106.
Selwyn, S. and Howitt, L. F.—(1962) *Lancet*, ii, 548.
Shimkin, M. B., Lucia, E. L., Oppermann, K. C. and Mettier, S. R.—(1953) *Ann. intern. Med.*, **39**, 1254.
Shimkin, M. B., Mettier, S. R. and Bierman, H. R.—(1951) *Ann. intern. Med.*, **35**, 194.
Shimkin, M. B., Opperman, K. C., Bostik, W. L. and Low-Beer, B. V. A.—(1955) *Ann. intern. Med.*, **42**, 136.
Shimkin, M. B., Oppermann, K. C., Low-Beer, B. V. A. and Mettier, S. R.—(1954) *Ann. intern. Med.*, **40**, 1095.
Stewart, A.—(1961) *Br. med. J.*, i, 462.
Stewart, A., Pennybacker, W. and Barber, R.—(1962) *Br. med. J.*, ii, 882.
Stewart, A. M. and Hewitt, D.—(1959) *Br. med. Bull.*, **15**, 73.
Tyrrell, D. A. J.—(1963) 'Mechanisms of virus infection'. London and New York (Academic Press), p. 248.
Videbaek, A.—(1947) 'Heredity in human leukaemia and its relation to cancer'. London (H. K. Lewis).
Wood, E.—(1960) *Br. med. J.*, i, 1760.
World Health Organisation.—(1959) *Tech. Rep. Ser. Wld Hlth Org.*, 170.